# PRotective Effect on the coronary microcirculation of patients with DIabetes by Clopidogrel or Ticagrelor (PREDICT): study rationale and design. A randomized multicenter clinical trial using intracoronary multimodal physiology

**DOI:** 10.1186/s12933-017-0543-5

**Published:** 2017-05-19

**Authors:** Enrico Cerrato, Alicia Quirós, Mauro Echavarría-Pinto, Hernan Mejia-Renteria, Andres Aldazabal, Nicola Ryan, Nieves Gonzalo, Pilar Jimenez-Quevedo, Luis Nombela-Franco, Pablo Salinas, Iván J. Núñez-Gil, José Ramón Rumoroso, Antonio Fernández-Ortiz, Carlos Macaya, Javier Escaned

**Affiliations:** 1 Unidad de Cardiología Intervencionista, Hospital Clínico San Carlos, 28040 Madrid, Spain; 2 Interventional Cardiology Unit, San Luigi Gonzaga University Hospital, Orbassano and Infermi Hospital, Rivoli, Turin, Italy; 3 Unidad de Cardiología Intervencionista, Hospital Galdakao, Bilbao, Spain

**Keywords:** Diabetes, IMR, CFR, FFR, Microcirculation, Ticagrelor

## Abstract

**Background:**

In diabetic patients a predisposed coronary microcirculation along with a higher risk of distal particulate embolization during primary percutaneous intervention (PCI) increases the risk of peri-procedural microcirculatory damage. However, new antiplatelet agents, in particular Ticagrelor, may protect the microcirculation through its adenosine-mediated vasodilatory effects.

**Methods:**

PREDICT is an original, prospective, randomized, multicenter controlled study designed to investigate the protective effect of Ticagrelor on the microcirculation during PCI in patient with diabetes mellitus type 2 or pre-diabetic status. The primary endpoints of this study aim to test (i) the decrease in microcirculatory resistance with antiplatelet therapy (Ticagrelor > Clopidogrel; mechanistic effect) and (ii) the relative microcirculatory protection of Ticagrelor compared to Clopidogrel during PCI (Ticagrelor < Clopidogrel; protective effect).

**Conclusions:**

PREDICT will be the first multicentre clinical trial to test the adenosine-mediated vasodilatory effect of Ticagrelor on the microcirculation during PCI in diabetic patients. The results will provide important insights into the prospective beneficial effect of this drug in preventing microvascular impairment related to PCI (http://www.clinicaltrials.gov No. NCT02698618).

**Electronic supplementary material:**

The online version of this article (doi:10.1186/s12933-017-0543-5) contains supplementary material, which is available to authorized users.

## Background

Type 2 diabetes mellitus (T2DM) is associated with a significant increase in the risk of coronary artery disease. As the prevalence of diabetes is estimated to double in the next 10 years, the burden of cardiovascular disease associated with this condition will dramatically increase. Although, over the last two decades, cardiovascular mortality has declined considerably in the general population, a similar trend has not been observed amongst diabetic patients [1]. This might be partly explained by the fact that diabetic patients have poorer outcomes than their non-diabetic counterparts [2, 3], both acutely and at long term follow up after coronary revascularization [4]. One of the mechanisms that may be related to this inferior outcome is the higher prevalence of periprocedural myocardial infarction (PMI) after percutaneous coronary intervention (PCI) observed in diabetic patients, which has been associated with endothelial dysfunction, pro-thrombotic state, chronic microvascular dysfunction, increased atheroma burden, vessel wall inflammation, and development of vulnerable plaques prone to distal embolization [5].

New antiplatelet agents, in particular Ticagrelor, might play a protective role in this setting. Ticagrelor, is different to Clopidogrel and other P2Y_12_ inhibitors, as it reduces the physiological clearance of adenosine by inhibiting its cellular uptake, thus increasing the plasma concentration of adenosine. As the primary aim of adenosine is achieving tonic and cellular protection during stress conditions [6], adenosine in turn may protect the myocardium from both ischemic and reperfusion injuries via its potent vasodilator effect and possibly by anti-inflammatory and antiplatelet properties [7].

Additionally, previous research [8] has identified a more pronounced effect of adenosine on microcirculatory resistance in patients with obesity and diabetes. Therefore, a potentially higher protective effect of Ticagrelor during PCI might be expected in this subgroup.

The PREDICT trial was therefore designed to investigate the potential protective effect of Ticagrelor on the coronary microcirculation during PCI in stable T2DM patients.

## Rationale

1. Coronary plaques at high risk for distal embolization during PCI [such as those with a thin cap fibroatheroma (TCFA)] are more prevalent in patients with T2DM [5]. Thus, this population is at high risk for developing myocardial injury and microcirculation impairment after PCI.
2. By blocking the ENT1 nucleoside cell membrane transporter, Ticagrelor inhibits cellular uptake of adenosine and thus its physiological clearance, increasing the circulating levels of adenosine. Adenosine may protect the myocardium from both ischemic and reperfusion injuries via its potent vasodilator effect and/or through its anti-inflammatory and antiplatelet properties [7]. Physiologically this may be recognized as a more pronounced decrease in coronary microvascular resistance induced by adenosine after the administration of Ticagrelor [9].
3. Previous research from our group [8] has suggested a more profound vasodilatory effect of adenosine on the microcirculation in patients with obesity and diabetes; these patients may have a greater protective benefit from Ticagrelor during PCI.

Therefore, Ticagrelor may be superior to Clopidogrel in providing microcirculatory protection during PCI procedures in patients with T2DM or pre-T2DM (primary hypothesis). Onset of treatment prior to PCI with Ticagrelor may be followed by a beneficial reduction in coronary microcirculatory parameters, as compared to Clopidogrel (secondary hypothesis).

## Methods

### Trial design and objectives

The PRotective Effect on the coronary microcirculation of patients with DIabetes by Clopidogrel or Ticagrelor (PREDICT) trial (http://www.clinicaltrials.gov No. NCT02698618) is a prospective, multicenter, randomized, open-label study designed to investigate the protective effect of Ticagrelor over Clopidogrel on the coronary microcirculation during PCI in patients with T2DM or pre-T2DM. The primary endpoint aims to document (i) a decrease in coronary microcirculatory resistance caused by treatment onset (Ticagrelor > Clopidogrel; mechanistic effect) and (ii) a lower increase in microcirculatory resistance caused by PCI (Ticagrelor < Clopidogrel; protective effect). A detailed list of endpoints is reported in Table 1 and defined in the Additional file 1: Appendix. A dedicated eCRF platform will be designed and hosted in the http://www.cardiogroup.org website.

**Table 1:** Study endpoints

Primary
Decrease in microcirculatory resistance caused by treatment onset (Ticagrelor > Clopidogrel)—*mechanistic effect*
Increase in microcirculatory resistance caused PCI (Ticagrelor < Clopidogrel)—*protective effect*
Secondary
Myocardial necrosis associated with PCI damage, assessed by cardiac biomarkers^a^
Absolute resistance value after PCI
Incidence of severe microcirculatory impairment defined as IMR > 29 after PCI
Subgroups analysis
Obese subjects

*PCI* percutaneous coronary intervention

^a^Third universal definition of myocardial infarction [40]

### Population recruitment and flow chart

The inclusion and exclusion criteria are listed in Table 2. The target population consists of patients with T2DM or pre-T2DM with stable ischemic heart disease and a single vessel stenosis or multiple vessels with single stenoses technically amenable to PCI and pressure wire study.

**Table 2:** Study inclusion and exclusion criteria

Inclusion criteria
Subject with type 2 diabetes mellitus or pre-type 2 diabetes mellitus status^a^
Subject must be older than 18 years
Written informed consent available
Documented silent ischemia, stable angina or patient who is scheduled for elective revascularization
Subject is eligible for PCI, and PCI target(s) have FFR ≤0.80
Exclusion criteria
Prior myocardial infarction in the territory of the target vessel
Akinesia or dyskinesia in subtended myocardial segments
Severe impairment of left ventricular function (LVEF <35%)
PCI target is a chronic total occlusion
Target lesion has been treated previously (restenotic lesions)
Target vessel is a saphenous vein graft or a surgical graft has been anastomosed to the target vessel
TIMI flow ≤1 prior to guide wire crossing
Subject is not eligible for treatment with drug eluting stent
Bleeding disorders or chronic anticoagulant treatment
Left main stenosis >50%
Coronary surgery deemed more beneficial for the patient than PCI
Ongoing treatment with Ticagrelor
Intolerance or contraindications to anti-platelet drugs
Contraindications for adenosine administration
Platelet count <75,000 or >700,000/mm^3^
Pregnant or breast feeding patient
History of intracranial hemorrhage
Severe hepatic impairment

*FFR* Fractional Flow Reserve, *LVEF* Left Ventricular Ejection Fraction, *PCI* percutaneous coronary intervention, *TIMI* thrombolysis in myocardial infarction

^a^2014 American Diabetes Association definition [41]

**Fig. 1:**
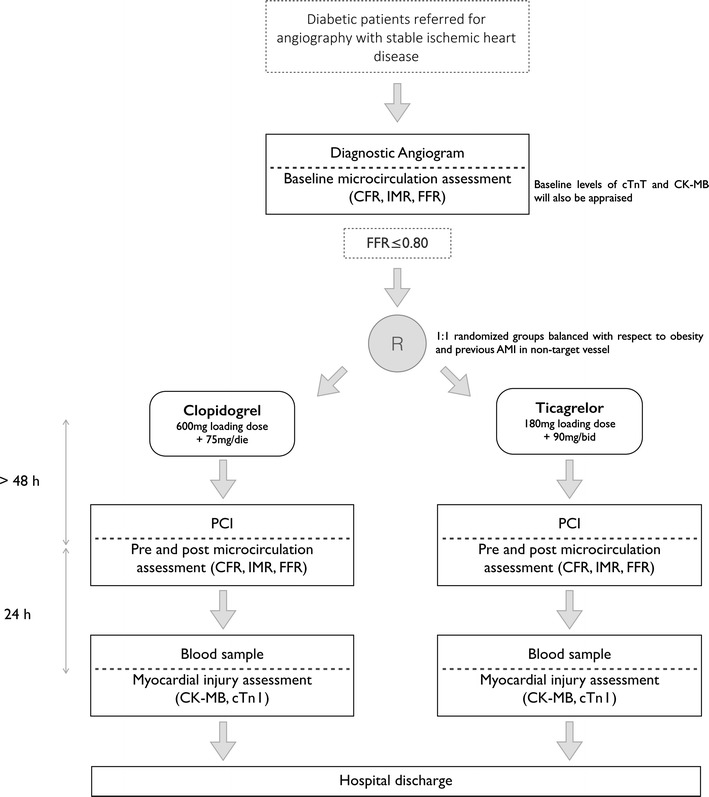
Study flow chart. *CFR* coronary flow reserve, *IMR* Index of Microvascular Resistance, *FFR* Fractional Flow Reserve, *AMI* acute myocardial infarction, *PCI* percutaneous coronary intervention, *CK*-*MB* creatine kinase myoband, *cTn I* cardiac troponin I

The study will be conducted as follows (Fig. 1, flow chart):

1. Patient identification and enrollment: All consecutive patients with stable ischemic heart disease and T2DM or pre-T2DM referred for coronary angiography will be screened as potentially eligible for the study. Assessment of coronary stenosis severity using Fractional Flow Reserve (FFR) and the status of the microcirculation including measurement of coronary flow reserve (CFR) and Index of Microvascular Resistance (IMR) [10, 11] will be performed with the same pressure guidewire as part of the diagnostic process. Revascularization will be considered whenever a FFR ≤0.80 is found in a stenosis amenable to PCI. Eligible patients requiring PCI will be informed of the characteristics of the study and invited to participate.
2. Randomization: Patients will be randomly assigned (1:1 ratio) to receive either Clopidogrel (600 mg loading dose followed by a daily dose of 75 mg) or Ticagrelor (180 mg loading dose followed by a dose of 90 mg b.i.d). The groups will be balanced according to the presence or absence of obesity [12] (Body Mass Index ≥ 30 kg/m^2^) [12] with the implementation of a dedicated randomization list. Patients who are already on oral treatment with Clopidogrel 75 mg/day are allowed to enter the protocol. According to randomization arm these patients will be assigned, after baseline assessment of microcirculation, to continue Clopidogrel 75 mg/day or be switched to Ticagrelor (180 mg loading dose followed by a dose of 90 mg b.i.d).
3. PCI procedure: PCI procedure will be deferred for at least 48 h after the first administration of the study drug treatment in order to allow approximately 5 mean-half life times of their active metabolites, similar to a previously published study [9].
  3.1. Pre-PCI: Multimodal physiological evaluation (FFR, CFR, IMR) will be repeated.
  3.2. PCI: For all patients undergoing PCI, unfractioned heparin will be administered at the time of PCI. The PCI procedures will be performed using standard techniques using and second generation Drug Eluting Stents. Balloon pre-dilatation will be mandatory before stent implantation using a semi-compliant balloon with a diameter smaller than 75% of the distal reference vessel size in order to avoid confounding effects related to pre-dilation [13]. Post-dilation will be performed according to clinical practice although it will not be mandatory. All PCI characteristics (materials and techniques) will be recorded.
  3.3. Post-PCI: After stent implantation, multimodal intracoronary physiological evaluation will be repeated (FFR, CFR, IMR).
4. In-hospital stay: High-sensitivity cardiac troponin and creatine kinase myoband (CK-MB) will be determined in blood samples taken after the intervention (8 and 24 h in all cases, and every 24 up to 72 h in case of rising myocardial markers).
5. In-hospital follow-up and data acquisition: Major adverse cardiac events as well as any bleeding complications occurring during in-hospital stay will be recorded (see Additional file 1: Appendix). All data required to test the primary and secondary endpoints will be collected before hospital discharge.
6. Length of participation: Patients will terminate their study participation at the time of hospital discharge.

### Pharmacological treatment(s)

Any concomitant medication used during the study will be recorded as well as any other required medication administered during hospitalization according to current guidelines and clinical practice. Any patients not taking long-term acetylsalicylic acid (ASA) before the procedure will receive a 300 mg oral dose of ASA at the time of inclusion in the study. All patients will continue their chronic treatment after the study termination as prescribed by their cardiologist. Dual antiplatelet therapy regime (DAPT) after discharge will be decided by the responsible physician according to current recommendations, bearing in mind the recommendations on DAPT switching if required [14, 15].

### Invasive multimodal physiology assessment

An intracoronary pressure and temperature sensor-tipped guidewire (St. Jude Medical) will be used to measure distal coronary pressure and the index of coronary flow derived from the coronary thermodilution method as previously described [11]. Three thermodilution curves will be obtained from a hand-held, 3 ml injection of room temperature saline, initially at baseline and then during maximal hyperemia, the latter achieved by an infusion of 140 µg/kg/min of adenosine via a large peripheral vein. Mean transit time (Tmn) at baseline and during maximal hyperemia will be derived from the thermodilution curves. Simultaneous recordings of mean aortic pressure (guiding catheter, Pa) and mean distal coronary pressure (distal pressure sensor, Pd) will be obtained at baseline and during maximal hyperemia. The coronary flow reserve (CFR) will be calculated from the ratio of hyperemic to baseline Tmn. The index of microcirculatory resistance (IMR) will be calculated using the following equation:

$$ {\text{IMR}} = {\text{Pa}} \times {\text{Tmn }}\left[ {\left( {{\text{Pd}} - {\text{Pw}}} \right)/\left( {{\text{Pa}} - {\text{Pw}}} \right)} \right], $$

where Pw is the coronary wedge pressure measured during vessel occlusion at the time of PCI. Calculation of IMR using Yong’s correction [16] (that does not require the incorporation of Pw) will be performed and reported. The FFR will be calculated from the ratio of distal to proximal pressures at maximal hyperemia.

## Sample size and statistical analysis

As no studies are currently available reporting on the impact of Ticagrelor on microvascular function compared to Clopidogrel, we based our sample size calculation on a previous study [10] showing that the intracoronary administration of Nicorandil after PCI resulted in a difference of 10 units in deltaIMR with respect to the control group. Assuming a difference of 10 in deltaIMR, in the experimental vs. control group, and a standard deviation of 10 a total of at least 20 patients per group would be needed to achieve an 80% power at a 2-sided alpha of 0.025, which accounts for the multiple primary endpoint design with a Bonferroni correction. Therefore, we aim to enroll 25 patients per group in order to take into account a 25% of potential missing data or major protocol violation due to clinical reasons (for example: clinical instability of patient between index and PCI procedure leading to urgent revascularization before completion of the protocol).

Any subject who took no trial medication will be eliminated from the full analysis set. Although a low or null proportion of exclusions due to treatment non-compliance is expected, any potential biases arising from this exclusion will be addressed. Imputation techniques will be used in an attempt to compensate for missing data. Regarding missing data, for primary endpoints, listwise deletion will be used meanwhile for the rest of analyses, listwise deletion or multiple imputation will be considered, depending on the type of missing process and on the quantification of the efficiency loss due to case deletion in each case. An investigation will be made concerning the sensitivity of the results of analysis to the method of imputation, especially if the number of missing values is substantial.

Being PREDICT a multicenter trial, the influence of the centre on the treatment effect and other endpoints will be addressed, and further adjustment will be done in the subsequent analysis, if necessary. Special attention will also be paid to the role of baseline measurements of the primary variable and diabetes treatment. Moreover, obese patients and those with previous myocardial infarction are subgroups of interest and their influence on the primary variables will be addressed and interpreted. However, subgroup analyses here have a exploratory nature, and therefore, interpreted cautiously.

A diagram of the participant flow showing for each group, the number of participants who were randomly assigned, received intended treatment, and were analyzed for the primary outcome will be provided, as recommended by the CONSORT 2010 statement.

Categorical variables will be reported as frequencies and percentages. Continuous variables will be expressed as mean ± SD or as median (Q_1_–Q_3_), according to the normality of their distribution, which will be tested with the Shapiro–Wilk test. Bartlett’s test will be performed to demonstrate homogeneity of variances between more than two groups. Primary endpoints will be assessed using the Student *t* test, if the samples are normally distributed or their variances are homogeneous; or Mann–Whitney U test, otherwise. P values will be corrected in order to account for the multiple endpoint. For the analysis of secondary variables, comparisons between continuous variables will be performed using the (paired or unpaired) Student *t* test or Mann–Whitney U test. The IMR at baseline, before PCI, and after PCI will be compared with an ANOVA for repeated measures or with the Friedman test, as appropriate. Comparisons between categorical variables will be evaluated using the Fisher exact test or the Pearson Chi square test, as appropriate. Correlations between continuous variables will be assessed using the Pearson or the Spearman rank correlation test. A 2-way ANOVA for repeated measures will be used to detect changes in IMR levels over time in the two study groups. Finally linear and logistic regression models will be carried out in order to identify predictors of periprocedural myonecrosis and delta-IMR. Analysis will be performed both in the full analysis set and a per protocol analysis and any differences between them would be the subject of explicit discussion and interpretation. In case of having multiple stenoses in some patient, additional adjusted analysis will be provided. Statistical analyses will be performed using the R statistical software [17] and p values <0.05 (2-tailed) will be considered significant. Whenever possible, estimates will be accompanied by confidence intervals.

## Participating centers

PREDICT will be coordinated by Hospital Clínico San Carlos (Madrid, Spain). Other three centers in Spain will participate in patient enrollment: Hospital de Galdakao (Bilbao), Hospital Universitario de Cabueñes (Gijón), Hospital Puerta de Hierro, Majadahonda.

## Discussion

The main purpose of the PREDICT trial is to test if Ticagrelor provides greater protection to the coronary microcirculation during PCI in diabetic patients compared to Clopidogrel as assessed by microvascular resistance indices. The results will provide important insights into the prospective beneficial effect of this drug in preventing microvascular impairment related to PCI. In the following paragraphs, we briefly discuss the rationale behind our study hypothesis.

### Microcirculatory damage during percutaneous coronary interventions

Instrumentation of atheromatous vessels during PCI, such as balloon dilation or stent implantation, may damage the subtended microcirculation and myocardium. The most dramatic presentation of this phenomenon is the so-called no-reflow phenomenon [18], in which coronary flow is interrupted or severely impaired despite the absence of epicardial obstruction, causing acute ECG and hemodynamic disturbances and a variable degree of myocardial damage. However, in most cases peri-procedural myocardial damage has a much more subtle presentation or is even clinically unnoticed. Distal embolization of particles released from the PCI target lesion constitutes the main cause of peri-procedural microcirculatory damage [19]. In stable patients, micro-emboli have an origin in atheromatous plaque, mainly derived from cholesterol rich deposits named atheromatous gruel. Several studies [20] using virtual histology Intra Vascular Ultrasound (VH-IVUS), frequency-domain optical coherence tomography (FD-OCT) and near infrared spectroscopy (NIRS) have linked the occurrence of the no-reflow phenomenon following PCI to cholesterol-rich plaques, mainly thin cap fibroatheromas. The same techniques have documented a higher prevalence of these types of plaques in patients with T2DM [5], who typically constitutes a higher-risk subset of patients for PCI treatment. Patients with diabetes often have comorbidities and a greater burden of coronary artery disease [21]. However, despite correction for these factors, diabetic patients still consistently have poorer outcomes than their non-diabetic counterparts especially in the setting of PCI [2, 3]. The abnormal coronary microcirculation [22] along with the higher risk of peri-procedural microcirculatory damage in T2DM represents one of the harmful and still unmet issues potentially connected with a poor long-term outcome. Additionally, even an optimal glycaemic control (HbA1c < 7%) does not predict a better coronary microcirculatory function in T2DM [23] claiming for a more appropriate strategies for prevention of coronary microvascular dysfunction.

Several authors have proposed pharmacological strategies targeting the microcirculation to prevent peri-procedural damage. As cardiac biomarker release is a vague indicator of microcirculatory damage (it may occur as a result of small side branch occlusion unnoticed in angiography), the use of physiological techniques to measure modifications in microcirculatory resistance (such as the IMR) has been advocated [11] as a surrogate of microcirculatory damage. IMR is reproducible, and mounting evidence supports its value as a meaningful diagnostic tool, particularly immediately after PCI. IMR values > 32 U (median) have been associated with higher infarction size (as assessed by CK-MB) and worse wall motion score at 3 months assessed by echocardiography. Moreover, IMR has been found to be the only significant predictor of recovery of LV function after ST Elevation Miocardial Infarction (STEMI) and previous studies conducted in T2DM patients demonstrated that post-PCI IMR is higher compared to non-T2DM individuals.

### P2Y_12_ receptors inhibitors: platelet reactivity and endothelial function

In patient with stable CAD, addition of Clopidogrel therapy results in an increase in endothelial function at the primary endpoint (assessed via reactive hyperaemic index) of 3 months. The improvement in endothelial function was already evident after 1 week of Clopidogrel therapy and seems to be not related to Clopidogrel effects on platelet aggregation [24].

On the other hand, among patients, those with T2DM exhibited increased platelet reactivity compared to patients without diabetes despite combined treatment with Clopidogrel and aspirin even when a loading dose of Clopidogrel rather than small daily doses was used [25]. The mechanisms leading to the high on-treatment platelet reactivity in T2DM patients are not fully elucidated and could potentially involved genetic factors [26].

Thus, after the introduction of new P2Y_12_ agents, the influence of Prasugrel or Ticagrelor on platelet reactivity in T2DM patients was object of studies demonstrating that Ticagrelor treated patients have overall lower platelet reactivity than patients on Prasugrel, independently of T2DM status or insulin treatment [27]. The effects of Prasugrel versus Clopidogrel on Coronary microvascular function in patients undergoing elective PCI was recently reported by Mangiacapra et al. [28] in the PROtecting MICROcirculation during coronary angioplasty (PROMICRO-2) trial. Compared with baseline, IMR increased post-PCI in the Clopidogrel group (p = 0.009), but not in the Prasugrel group (p = 0.299). Despite some limitations (small sample size; single time point measurement of IMR post-PCI; lack of intracoronary imaging for assessment of plaque burden) the result of this trial suggest that more intensive anti-platelet regimens might offer additional benefit compared with Clopidogrel also in the setting of elective PCI.

An ongoing randomised, prospective, controlled study [29] are also testing the effect of Clopidogrel, Prasugrel and Ticagrelor on multiple parameters of vascular function, platelet aggregation, oxidative and inflammatory stress before and up to 1 month after coronary artery stenting.

### Ticagrelor and adenosine metabolism

Beyond the antagonizing effect on P2Y_12_ receptors and the improvement in microvascular endothelial function that we reported above, Ticagrelor may also protect microcirculation increasing the circulating levels of adenosine, Several studies have consistently shown that Ticagrelor inhibits the cellular uptake of adenosine. Intracellular adenosine is rapidly uptaken and metabolized to inosine by adenosine deaminase or transformed into adenine nucleotides by adenosine kinase [7] and therefore by inhibiting its transport into cells its half-life can be increased. Ticagrelor achieves this by inhibiting the sodium-independent nucleoside transporter 1 (ENT1) [30] leading to significantly conserved adenosine levels in human whole blood in vitro experiments. Finally, a wide spectrum of biological effects and physiological responses are carried out through a pathway mediated by adenosine interaction with at least four different receptor subtypes (A1R, A2AR, A2BR, and A3R) which are coupled to stimulatory or inhibitory G proteins [31].

Wittfeldt et al. [9] first demonstrated an adenosine related mode of action for Ticagrelor in humans. In a double-blind, placebo-controlled, crossover study Coronary Blood Flow Velocity (CBFV) was measured using transthoracic Doppler echocardiography at rest after multiple stepwise adenosine infusions. Ticagrelor increases the adenosine-induced physiological responses as shown by an increased area under the curve (AUC) for CBFV response compared to placebo. This increase correlated with plasma Ticagrelor concentrations and was mediated by adenosine receptors with a reversal of this effect after infusion of theophylline, a non-selective competitive adenosine receptor antagonist.

### Protective effects of adenosine on myocardial injury associated with percutaneous interventions or acute coronary syndromes

Adenosine is routinely used in the catheterization laboratory for the treatment of the no-reflow phenomenon during PCI, which constitutes an extreme manifestation of peri-procedural damage. No-reflow develops dramatically in response to vessel instrumentation, with contrast medium stagnation in epicardial arteries, persistent myocardial blush and, frequently, accompanying ECG and hemodynamic changes. This complication is the result of plugging of the coronary microcirculation by downstream embolization of micro-thrombi or atheroma dislodged from the culprit lesion as a result of its manipulation during PCI [32]. Reperfusion injury may also manifest as no-reflow phenomenon. A protective effect of adenosine administration in preventing ischemia/reperfusion injury has been demonstrated in both humans [33] and animal models [34].

The effects of adenosine on no-reflow have been investigated in numerous studies. A recent meta-analysis of seven randomized clinical trials supports the benefit of intracoronary adenosine in terms of post-PCI ST-segment resolution and reduced residual ST-segment elevation [35]. Additionally, the PROMISE trial recently showed a reduction in infarct size in patients undergoing administration of high-dose intracoronary adenosine [36]. These effects may be related to the potent vasodilatory effects and potential anti-inflammatory and antiplatelet properties of adenosine. Finally, the CV-TIME trial [37] recently demonstrated that in patients with STEMI treated by primary PCI, a 180 mg loading dose of Ticagrelor might be more effective in reducing microvascular injury than a 600 mg loading dose of Clopidogrel, as demonstrated by IMR immediately after primary PCI.

### Microcirculatory and systemic responses to adenosine

The response after adenosine administration is heterogeneous and associated with relevant differences in clinical and intracoronary physiological characteristics. Based on a study performed with intracoronary multimodal physiology, we recently reported that patients with T2DM or the metabolic syndrome [8] demonstrate enhanced responses to adenosine both at a systemic and coronary microcirculatory level (as shown by a drop in systemic blood pressure and microcirculatory resistance). A possible explanation for this observation may be related to the heterogeneous impairment in adenosine receptor subtypes A1 reported in obese humans compared to non-obese [38].

This finding supports the hypothesis that the myocardial protective effect of Ticagrelor may be higher in patients with T2DM or the metabolic syndrome. This is of particular importance, as PCI in patients with diabetes has been associated with higher peri-procedural events than in non-diabetic patients.

## Conclusions

The role of adenosine in protecting the microcirculation remains controversial [39]. This property of adenosine may be particularly important in the context of high-risk patients such as diabetics. PREDICT will be the first randomized multicenter clinical trial to test the adenosine-mediated vasodilator effect of Ticagrelor on the microcirculation during PCI in diabetic patients. The results will provide important insights into the prospective beneficial effect of this drug in preventing microvascular impairment related to PCI.

## Additional file

**Additional file 1: Appendix.** Definitions and outcomes.

## Abbreviations

AMI: acute myocardial infarction
AUC: area under the curve
BMI: Body Mass Index
CBFV: Coronary Blood Flow Velocity
CFR: coronary flow reserve
CK-MB: creatine kinase myoband
C Tn I: cardiac troponin I
DAPT: dual antiplatelet therapy
ECG: 12-leads electrocardiogram
ENT1: sodium-independent nucleoside transporter 1
FD-OCT: frequency-domain optical coherence tomography
FFR: Fractional Flow Reserve
FPG: fasting plasma glucose
GFR: glomerular filtration rate
HbA1c: glycosylated hemoglobin
HDL: high density lipoprotein cholesterol
IFG: impaired fasting glucose
IMR: Index of Microvascular Resistance
IVUS: Intra Vascular Ultrasound
LDL: low density lipoprotein cholesterol
LVEF: Left Ventricular Ejection Fraction (%)
MACEs: major cardiovascular events
NIRS: near infrared spectroscopy
OGTT: oral glucose tolerance test
PCI: percutaneous coronary intervention
STEMI: ST Elevation Miocardial Infarction
T2DM: type 2 diabetes mellitus
TCFA: thin-cap fibroatheroma
TIMI: Thrombolisis in Myocardial Infarction Flow
VH-IVUS: virtual histology Intra Vascular Ultrasound
WHO: World Health Organisation
