# Supplementary material for: PRotective Effect on the coronary microcirculation of patients with DIabetes by Clopidogrel or Ticagrelor (PREDICT): study rationale and design. A randomized multicenter clinical trial using intracoronary multimodal physiology
Source: Cardiovasc Diabetol. 2017 May 19;16:68. doi: 10.1186/s12933-017-0543-5 (PMC5438565; doi:10.1186/s12933-017-0543-5)
Supplement: Supplementary file 1 — Additional file 1: Appendix. Definitions and outcomes. [file 12933_2017_543_MOESM1_ESM.doc]

APPENDIX

**Definitions**

**Index of microcirculatory resistance (IMR)** will be calculated using the following equation: IMR = Pa x Tmn[(Pd - Pw )/(Pa - Pw)],. where Pw is the coronary wedge pressure measured during vessel occlusion at the time of PCI. Calculation of IMR using Yong´s correction (that does not require the incorporation of Pw) will be performed and reported

**IMR at baseline** will be defined as the value of IMR registered during the diagnostic angiogram.

**IMR Pre-PCI** will be defined as the value of IMR registered after randomization just before performing PCI and stenting.

**IMR Post-PCI** will be defined as the value of IMR registered just after performing PCI and stenting.

**Delta IMR Post-PCI** will be defined as the absolute difference in the IMR value associated to PCI [“Delta IMR Post-PCI” = (IMR value post-PCI) minus (IMR value pre-PCI)].

**Delta IMR Pre-PCI** will be defined as the absolute difference in the IMR value associated to PCI [“Delta IMR Pre-PCI” = (IMR value pre-PCI) minus (IMR value at baseline)].

**Diabetes Mellitus Type II**: according to the 2014 American Diabetes Association Diagnosis and classification of diabetes mellitus (30) patient will be considered to be diabetic if any of the following criteria is met:

1. haemoglobin A1C ≥ 6.5% The test should be performed in a laboratory using a method that is NGSP certified and standardized to the DCCT assay.* or
2. FPG ≥126 mg/dL (7.0 mmol/L). Fasting is de fi ned as no caloric intake for at least 8 h.* or
3. Two-hour plasma glucose ≥200 mg/dL (11.1 mmol/L) during an OGTT. The test should be performed as described by the World Health Organization, using a glucose load containing the equivalent of 75 g anhydrous glucose dissolved in water.* or
4. In a patient with classic symptoms of hyperglycemia or hyperglycemic crisis, a random plasma glucose 200 mg/dL (11.1 mmol/L).

*In the absence of unequivocal hyperglycemia, criteria 1–3 should be confirmed by repeat testing.

**Pre-diabetes:** according to American Diabetes Association (30) a patient will be considered to be in the pre-diabetic range if have:

1. an impaired fasting glucose (IFG) [fasting plasma glucose (FPG) levels 100 mg/dL (5.6 mmol/L) to 125 mg/dL (6.9 mmol/L)],
2. an impaired glucose tolerance (IGT) [2-h values in the oral glucose tolerance test (OGTT) of140 mg/dL (7.8 mmol/L) to 199 mg/dL(11.0 mmol/L)].

Individuals with IFG and/or IGT have been referred to as having prediabetes, indicating the relatively high risk for the future development of diabetes. IFG and IGT should not be viewed as clinical entities in their own right but rather risk factors for diabetes as well as cardiovascular disease.

**Obesity:** will be defined as a Body Mass Index ≥30 kg/m 2 according to the international classifications of the World Health Organisation (WHO).

**MACE:** Major adverse cardiac events will be defined as cardiac death, non-fatal myocardial infarction, and ischemia driven premature target lesion revascularization during hospitalisation.

**PCI-related myocardial infarction (type 4a)** will be defined as post- procedural increase in Troponin more than 5 times the 99th percentile of the upper reference limit for patients with baseline negative myocardial necrosis markers consistent with the joint European Society of Cardiology/American College of Cardiology Foundation/American Heart Association/World Heart Federation Task Force for Universal Definition of Myocardial Infarction consensus statement on the definition of myocardial infarction for clinical trials on coronary intervention. PCI-related myocardial infarction was also reported in the study results as increase in CK-MB more than 3 times the 99th percentile of the upper reference limit.

**Death**: any cause death.

**Cardiac death**: any death without a non-cardiac cause.

**Repeat revascularization**: classified as target lesion re-interventions (TLR) inside the implanted stent or within 5 mm proximally or distally or repeated interventions in the same vessel (TVR) by percutaneous coronary interventions (PCI) or by coronary artery bypass graft surgery.

**Stent thrombosis (ST)** will be classified as “acute”- within 24 hours from the procedure, “sub-acute” up to 30 days, “late” from 30 days till 1 year and “very late” after 1 year after index procedure.

Thrombosis will be classified as definite, probable and possible according to the definition of Academic Research Consortium.

ST will be defined as the occurrence of one of the following events:

1. Angiographic documentation of complete or partial stent occlusion and target vessel related acute clinical ischemic event.

2. Autopsy documentation of complete or partial thrombotic stent occlusion

3. Myocardial infarction in the distribution of the stented vessel.

We will separately evaluate the incidence of possible ST by including all unexplained death after 30 days.

**Major bleeding** defined as the cumulative occurrence of intracranial or intraocular bleeding, hemorrhage at the vascular access site requiring intervention, a reduction in hemoglobin levels of at least 5 grams per deciliter, reoperation for bleeding or transfusion of a blood product (at least 2 units). All other bleeding events were considered as minor (i.e. epistaxis, blood traces in the stool, etc.

**Clinical and Technical Procedures**

|  | Enrollment, diagnostic procedure and randomization | PCI procedure | Discharge |
| --- | --- | --- | --- |
| Patient History |  |  |  |
| Physical Examination |  |  |  |
| Electrocardiography (ECG) |  |  |  |
| Blood Samples and Parameters Assessed |  |  |  |
| Invasive Multimodal Physiology Assessment |  |  |  |
| PCI with stenting |  |  |  |

Table: clinical and technical procedure timetable

Patient History

The patient‘s history must be taken according to the schedule as derived from the CRF with particular focus on major adverse cardiac events.

Physical Examination

The aim of the physical examination is to assess angina status, clinical history, and baseline medication. A complete physical examination of the patient must be performed before enrollment, after the procedure, and at any time when scheduled or unscheduled follow-up will be performed.

Electrocardiography (ECG)

All patients will undergo pre-intervention and post-intervention 12-lead ECG to detect procedure related ischemic changes (Q-wave myocardial infarction: appearance of a new pathological Q-wave). Calibration marks or clear notations should be inscribed on each ECG tracing to enable the interpreter to determine the paper speed and gain settings used in recording. Standard settings of a paper speed of 25 or 50 mm per sec according to the local routine, and a calibration of 10 mm per mV should be used unless required by technical reasons and indicated on the tracing.

Blood Samples and Parameters Assessed

Routine laboratory parameters must be assessed prior to the intervention as a part of the screening procedure in order to verify the enrolment criteria. Cardiac markers (CK-MB -creatinine phosphokinase myocardial bound and troponin) must be obtained in the morning following the procedure and, in case of values beyond normal, additionally after at least six hours until the normal range is reached again.

Parameters to be assessed before the procedure/randomization:

- Cardiac enzymes: CK-MB and troponin.

Parameters to be assessed after any catheterization procedure:

- Cardiac enzymes: CK-MB and troponin.

Timing of Blood Draws

The basic assessment is done before the index procedure or at time of randomization.

In case of significant elevation of cardiac enzymes, increase in serum creatinine, or drop in hemoglobin, a repeat evaluation should be performed until the peak of change in parameters can be assessed. Blood analysis will be repeated after any catheterization procedure. All blood parameters will be determined at the local centre laboratory of each participating hospital.

Treatment

- 1. Identity of products

Table 1. Identity of products

| Investigational product | Dosage form and strength | Manufacturer |
| --- | --- | --- |
| Clopidogrel | 75mg/day | Sanofi Winthrop Industrie |
| Ticagrelor (Brilinta) | 90mg/bid | ASTRAZENECA |

- 1. Doses and treatment regimens

Clopidogrel: loading dose of clopidogrel 600mg per os at the time of the randomization followed by 75 mg once a day per os according to current recommendations.

Ticagrelor: loading dose of ticagrelor 180 mg per os at the time of the randomization followed by ticagrelor 90 mg twice a day per os according to current recommendations.
